# Dual Silencing of Hsp27 and c-FLIP Enhances Doxazosin-Induced Apoptosis in PC-3 Prostate Cancer Cells

**DOI:** 10.1155/2013/174392

**Published:** 2013-06-19

**Authors:** Sang Soo Kim, Hee-Ju Cho, Jeong-Man Cho, Jung Yoon Kang, Hyun-Won Yang, Tag Keun Yoo

**Affiliations:** ^1^Eulji Medi-Bio Research Institute, Seoul 139-871, Republic of Korea; ^2^Department of Urology, College of Medicine, Eulji University, Hangeulbiseok-gil, Hagye-dong, Nowon-ku, Seoul 139-871, Republic of Korea; ^3^Department of Biotechnology, Seoul Women's University, Seoul 139-871, Republic of Korea

## Abstract

We evaluated effect of dual gene silencing of Hsp27 and c-FLIP in doxazosin-induced apoptosis of PC-3 cell. After transfection using Hsp27 and c-FLIP siRNA mixture (dual silencing), doxazosin treatment was done at the concentrations of 1, 10, and 25 **μ**M. We checked apoptosis of PC-3 cells with and TUNEL staining. We also checked interaction between Hsp27 and C-FLIP in the process of apoptosis inhibition. Spontaneous apoptotic index was 5% under single gene silencing of Hsp27 and c-FLIP and 7% under dual silencing of Hsp27 and c-FLIP. When doxazosin treatment was added, apoptotic indices increased in a dose-dependent manner (1, 10, and 25 **μ**M): nonsilencing 10, 27, and 52%; Hsp27-silencing: 14, 35, and 68%; c-FLIP silencing: 21, 46, and 78%; dual silencing: 38, 76, and 92%. While c-FLIP gene expression decreased in Hsp27- silenced cells, Hsp27 gene expression showed markedly decreased pattern in the cells of c-FLIP silencing. The knockout of c-FLIP and Hsp27 genes together enhances apoptosis even under 1 **μ**M, rather than low concentration, of doxazosin in PC-3 cells. This finding suggests a new strategy of multiple knockout of antiapoptotic and survival factors in the treatment of late-stage prostate cancer refractory to conventional therapy.

## 1. Introduction

Advanced prostate cancers eventually progress to the terminal stage of castration refractory prostate cancer (CRPC) which is not responsive to most of treatment modalities. Docetaxel-based chemotherapy has been the mainstay of treatment for this metastatic CRPC, and recently, other anticancer drugs such as cabazitaxel or abiraterone acetate are allowed for use, but their therapeutic benefits are still not that sufficient [1–4]. 

Researchers consider some defects in apoptotic signaling pathway or abnormal overexpression of antiapoptotic factors as the main causes of treatment resistance in patients with late-stage prostate cancer [5].

Among those antiapoptotic factors, bcl-2 [6], clusterin [7], heat shock prostein 27 (Hsp27) [8, 9], cellular-FLICE inhibitory protein (c-FLIP) [10], and GRP78 [11] have been widely reported. Overexpression of these factors could be induced by androgen deprivation therapy (ADT), chemotherapy, or other extreme stresses.

Recently, new therapeutic ways of blocking these factors are being developed as new drugs, and among them, clusterin ASO is now on Phase 3 clinical trial [12]. While knockdown of each factor alone can exert apoptosis inducing effect, blocking several factors which have some different pathways together may enhance apoptosis in prostate cancer cells. This concept can be applied to the development of new therapy against prostate cancer.

Hsp27, one of small Hsps, inhibits key effectors of the apoptotic pathway at the pre- and postmitochondria levels [13]. In prostate cancer, Hsp27 is associated with pathologic stage, Gleason score, lymph node metastasis, shorter biochemical recurrence, and poor clinical outcome [14, 15]. 

The c-FLIP is an inhibitor of apoptosis downstream of the death receptors Fas, DE4, and DR5 [16]. The expression of c-FLIP is closely related to the resistance to tumor necrosis factor-related apoptosis-inducing ligand (TRAIL) and FAS-mediated apoptosis in prostate and bladder cancers [17–19]. Therefore, c-FLIP is regarded as a new therapeutic target for relevant cancers [20]. 

Dual silencing is reported to be effective in augmenting biologic effect on laboratory level and technically feasible [21].

Doxazosin, an quinazoline derivative *α*1 adrenoreceptor antagonist, has been known to exert antitumor effect via induction of apoptosis in PC-3 cancer cells [22]. Doxazosin induces apoptosis via not an *α*1-adrenoceptor-dependent action but a death receptor-mediated pathway [23].

In this present study, we investigated the enhanced effect of double knockout of Hsp27 and c-FLIP genes using siRNA technology in PC-3 prostate cancer cells and also tried to find out whether there is any interactive role between the 2 factors by observing the expression of one factor under silencing of the other factor.

## 2. Materials and Methods

### 2.1. Cell Lines

PC-3 cells obtained from American Type Culture Collection (Bethesda, MD, USA) were maintained in F-12 medium. We compared as group treated scrambled siRNA, AI-LNCaP-scr-siRNA.

### 2.2. Doxazosin Treatment

Doxazosin (Sigma Aldrich Korea, Seoul, Korea) was prepared as described in a previous study [7]. Cultures at 80% confluence were changed to fresh media and treated with doxazosin or serum-free media containing 0.25% DMSO as control. 

### 2.3. Transfection with siRNA

The mRNA target sequences to Hsp27 (GeneBank Accession no. X54079.1) and c-FLIP (Gene ID: 8837) were designed using a siRNA template design tool (Ambion, Austin, TX, USA), and siRNA was prepared with a Silencer siRNA construction kit (Ambion). Three oligonucleotides Hsp27-1 (5′-GACCUACCGAGGAGCUUUCdTT-3′), Hsp27-2 (5′-UCGAGGCCCUGUAACUUG-3′), and Hsp27-3 (5′-CAGUAGUUCGGACAAACGAAGA-3′) were designed based on the publicly released Hsp27 DNA sequence and another three oligonucleotides FLIP-1, FLIP-2, and FLIP-3 designed for c-FLIP. The siRNAs were transfected into PC-3 cells with Lipofectamine 2000 (Invitrogen) employing 50 nM in 250 *μ*L Opti-MEM medium/60 mm culture dish. The transfected cells were allowed to grow for 24, 48, and 72 h at 37°C in a 5% CO_2_ incubator.

### 2.4. Total RNA Extraction, Conventional RT-PCR, and Real-Time RT-PCR

Total RNA was extracted using the TRIzol method (Invitrogen, Carlsbad, CA, USA). Cells (5.0 × 10^5^) were mixed in a test tube with 1 mL TRIzol solution. Prepared RNA was denaturated at 65°C for 15 min in a volume of 30 *μ*L and cooled on ice for at least 1 min. 2.0 *μ*g of denatured RNA were then annealed by addition of reaction mixture to a total volume of 20 *μ*L (4.0 *μ*L of 5× RT buffer, 10 pmol of primers, 2.0 *μ*L of 25 mM MgCl_2_, 2.0 *μ*L of 10 mM dNTPs, and 0.2 *μ*L of 1 M DTT in nuclease-free water) and incubated at 42°C for 70 min. The reaction was terminated at 95°C for 5 min, chilled on ice for 5 min and collected by brief centrifugation. To remove RNA, 1 *μ*L of RNase H was added to each tube followed by incubation at 37°C for 20 min. 1 *μ*L of cDNA was used for each PCR reaction.

PCR was performed with an SLAN real-time PCR detection system (LG Life Science, Korea) and SYBR Green reagents (Invitrogen, Carlsbad, CA, USA). Specific primers for human GAPDH, Hsp27, and c-FLIP were designed to work in the same cycling conditions (50°C for 2 min to permit uracil *N*-glycosylase cleavage, 95°C for 10 min, followed by 40 cycles of 95°C for 15 s, and 60°C for 1 min). The specificity of the nucleotide sequences chosen was confirmed by conducting basic local alignment search tool searches. We used 1.0 *μ*L of the reverse transcriptase product for PCR in a final volume of 25 *μ*L.

### 2.5. Western Blot Analysis

Preparation of total cell lysate and the procedures for Western blot analyses were performed essentially as described previously [16]. The antibody against c-FLIP was purchased from Santa Cruz Biotechnology (Santa Cruz, CA, USA). Antibody for Hsp27 was purchased from Millipore (Millipore, MA, USA). The quantity of the applied protein was normalized with anti-actin polyclonal antibody (Sigma Aldrich Korea, Seoul, Korea).

Samples with equal amounts of protein (20 *μ*g) from lysates of cultured PC-3 cells were subjected to SDS-PAGE and then transferred to a PVDF filter. The filters were blocked in TBS containing 5% nonfat milk powder at 4°C overnight and then incubated with each of diluted primary antibodies (Actin: 1 :  10,000; Hsp27: 1 : 1,000; c-FLIP: 1 : 2,000; Santa Cruz, CA, USA) for 1 hour. 

### 2.6. Immunofluorescence and TUNEL Staining

Cells on coverslips were rinsed with 1× phosphate-buffered saline (PBS) and then fixed with ice-cold methanol for 15 min. Samples were further permeabilized with PBS containing 0.025% Triton-X detergent (1× PBS-TX) for 10 min and blocked with 3% BSA in 1× PBS for 30 min. Cells were incubated with each of the primary antibodies (Hsp27: 1 : 100; c-FLIP: 1 : 50; Santa Cruz, CA, USA) for 1 hour at room temperature. Cells were washed 3 times for 5 mins with 1× PBS-TX and then incubated with green fluorescent- (FITC-) conjugated secondary antibodies (goat anti-mouse IgG and goat anti-rabbit IgG, Santa Cruz, CA, USA). Nuclei were counterstained with Hoechst 33258 (Sigma Chemical, St. Louis, MO, USA).

For TUNEL assays, fixed cells were incubated with an equilibrium buffer for 5 min using the in situ apoptosis detection kit, Fluorescein (ApopTag; Roche, BMS), and then treated in reaction buffer with 10 units of terminal deoxynucleotidyl transferase and 1 unit of deoxyuridine triphosphate-digoxigenin at 37°C for 1 hour. The reaction was terminated by adding stop/wash buffer and then washed twice with Tris buffer. Antidigoxigenin-FITC was added and reacted at 37°C for 30 min. After washing with distilled water, nuclei were counterstained with Hoechst 33258 (Sigma Chemical, St. Louis, MO, USA), and apoptosis in the cells was observed under a fluorescent microscope. Cells with green fluorescent (FITC) colored nuclei were considered apoptotic. For quantifying apoptotic cells, apoptotic and total cells were counted in 5 random fields scoring between 300 and 500 cells, and the numbers of apoptotic cells were expressed as percentages of the total cell population. Immunofluorescent staining slides and TUNEL staining slides were observed with microscope (TE-300, Nikon, Japan).

### 2.7. Statistical Analysis

Data are expressed as mean ± SD or median (interquartile range). 

## 3. Results

### 3.1. Assessment for Hsp27 and c-FLIP RNA Interference with siRNAs in PC-3 Cells

**Figure 1:**
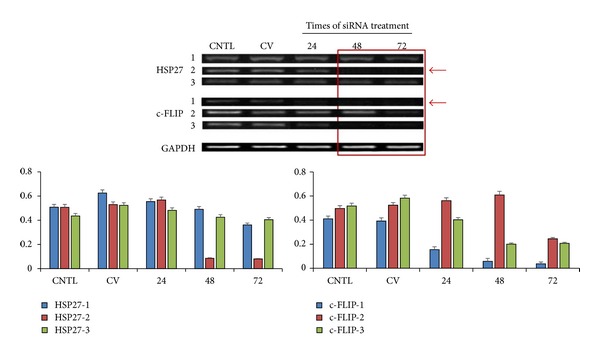
RT-PCR bands show effective silencing of the heat shock protein 27 (Hsp27) and c-FLIP gene expression in PC-3 cells after small interfering RNA (siRNA) treatment.

For downregulation of Hsp27 expression in PC-3 cells, three different Hsp27-specific siRNAs (Hsp27-1, Hsp27-2, and Hsp27-3) were used for transfection studies. As a control, cells were transfected with siRNA against scrambled sequence. To determine the efficiency of the downregulation of Hsp27 expression in PC-3 cells, mRNA levels of Hsp27 were counted by RT-PCR. Forty-eight hours after transfection, Hsp27-2 siRNA downregulated Hsp27 mRNA level to approximately 17% of control level. The downregulation of Hsp27 expression caused by Hsp27-2 siRNA-mediated silencing was maintained until 72 hours (Figure 1). 

Similar to Hsp27, three kinds of siRNAs (c-FLIP-1, c-FLIP-2, and c-FLIP-3) were used for c-FLIP silencing in PC-3 cells. c-FLIP-1 siRNA downregulated c-FLIP mRNA level to approximately 14% of control level after 48 hours of transfection. Among three siRNAs, The c-FLIP-1 siRNA-mediated silencing decreased c-FLIP mRNA expression until 72 hours (Figure 1).

**Figure 2:**
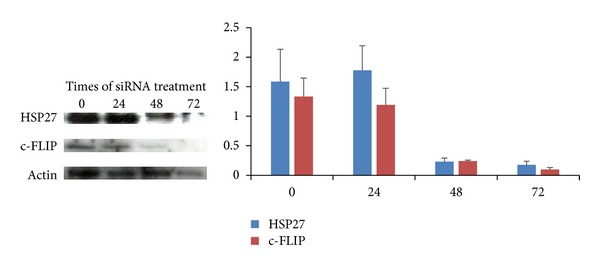
Immunoblotting shows effective silencing of the heat shock protein 27 (Hsp27) and c-FLIP in PC-3 cells after siRNA treatment.

Endogeneous expression of Hsp27 in Hsp27-2 siRNA-transfected PC-3 cells was reduced approximately to 14.6% of control level after 48 hours after transfection when measured by Western blot analysis with an anti-Hsp27 antibody (Figure 2). Likewise, Western blot analysis for anti-c-FLIP antibody also revealed the reduction of endogeneous c-FLIP expression in c-FLIP-1 siRNA-transfected PC-3 cells in 18.7% after 48 hours and maintained until 72 hours (Figure 2).

### 3.2. TUNEL Analysis

**Figure 3:**
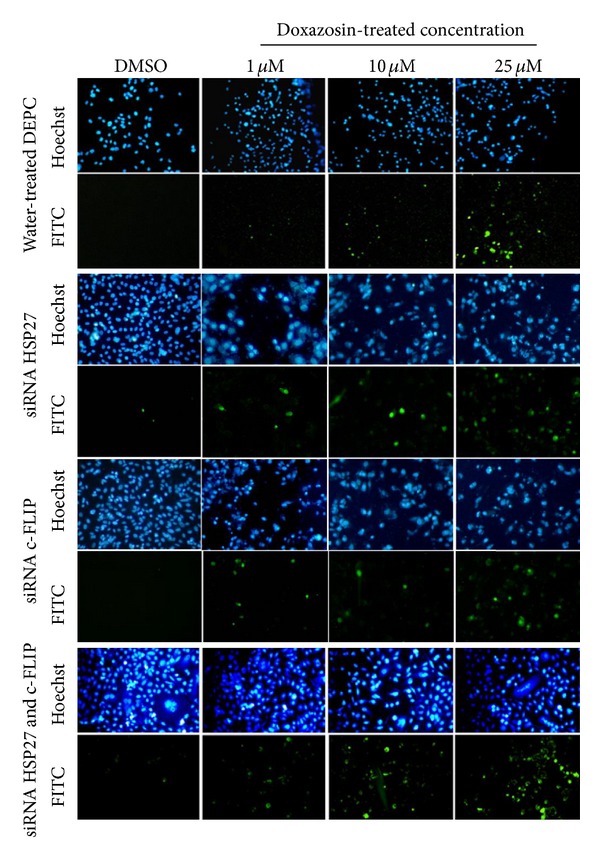
In situ detection of apoptotic cells in siRNA (Hsp27, c-FLIP, dual) transfected PC-3 cells after 48 hours at each dosage of doxazosin treatment (1, 10, and 25 *μ*M). In situ detection of apoptotic cells in prostate cancer cells was performed by 3′-end labeling with digoxigenin-dUTP using terminal transferase.

Figure 3 shows the results of Hoechst and TUNEL fluorescent staining for nuclear morphology and patterns of apoptosis in Hsp27 and c-FLIP gene silenced PC-3 cells when treated with doxazosin (1, 10, and 25 *μ*M) for 24 hours. Nuclear condensation and fragmentation, characteristic findings of apoptosis, were found in TUNEL-positive cells. 

In the cells without siRNA transfection, the number of TUNEL-positive cells was minimal under 1 *μ*M of doxazosin treatment, but it increased gradually in a dose-dependent manner, and finally a significant number of apoptotic bodies were observed under 25 *μ*M of doxazosin treatment.

Compared to the nonsilenced cells, in the cells transfected with either siRNA targeting Hsp27 or c-FLIP genes, the numbers of TUNEL-positive cells were increased in all concentrations of doxazosin. And when they were transfected with both siRNAs targeting Hsp27 and c-FLIP gene together, the number of TUNEL-positive cells was increased more significantly in all concentrations of doxazosin. In this group of cells, TUNEL-positive cells were visible quite a lot even with 1 *μ*M of doxazosin treatment.

**Figure 4:**
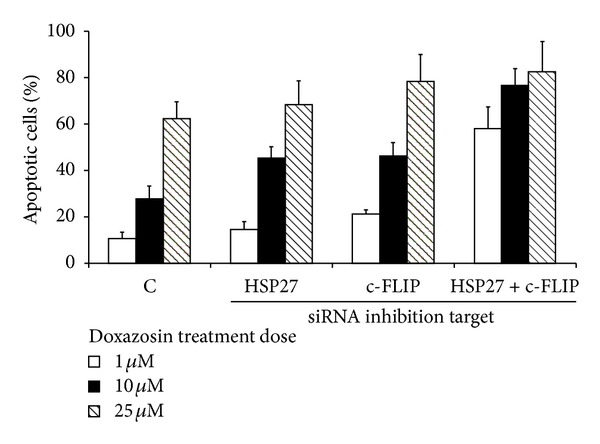
Apoptotic indices in siRNA (Hsp27, c-FLIP) transfected cells after 48 hours at each dosage of doxazosin treatment (1, 10, and 25 *μ*M).

Spontaneous apoptotic index was 5% under single gene silencing of Hsp27 or c-FLIP and 7% under dual silencing of Hsp27 and c-FLIP genes together. When doxazosin treatment was added, apoptotic indices increased in the dose-dependent manner (1, 10, and 25 *μ*M): nonsilencing 10, 27, and 52%; Hsp27 silencing: 14, 35, and 68%; c-FLIP silencing: 21, 46, and 78%; dual gene silencing: 38, 76, and 92% (Figure 4). Annexin V staining showed similar findings (data not shown).

### 3.3. Cross-Checking for Hsp27 and c-FLIP RNA Interference with siRNA for PC-3

**Figure 5:**
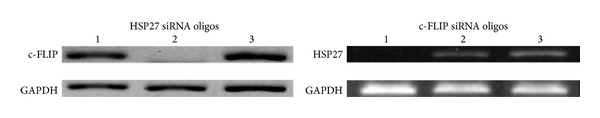
RT-PCR band of small interfering RNA (siRNA) treatment of the heat shock protein 27 (Hsp27) and c-FLIP after 48 hours in PC-3 cells.

**Figure 6:**
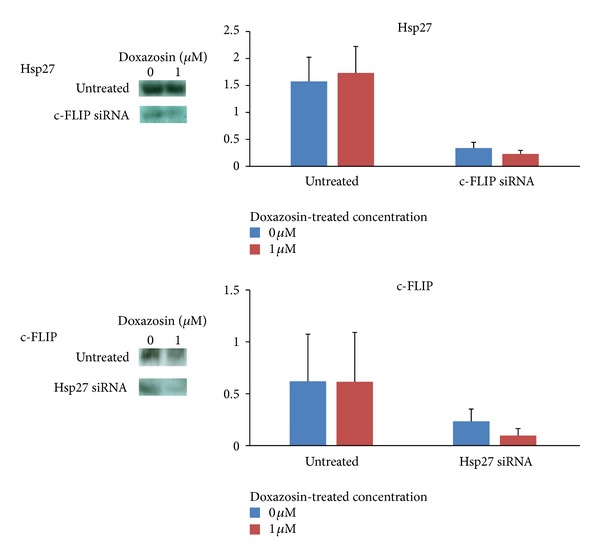
Electrophoretogram of immunoblot for Hsp27 and c-FLIP expressions at the PC-3 cells.

The interaction between 2 factors is investigated by observing the expression of one factor under silencing of the other factor. When Hsp27 is silenced successfully, c-FLIP gene expression was suppressed, and when c-FLIP was inhibited by siRNA transfection, Hsp27 gene expression was also downregulated (Figure 5). Similar findings were observed in protein level. Western blot analysis showed that when c-FLIP was inhibited by siRNA transfection, Hsp27 protein expression was downregulated, and protein expression of c-FLIP was suppressed when Hsp27 gene was silenced. Addition of 1 *μ*M of doxazosin enhanced downregulation of c-FLIP protein expression induced by Hsp27 gene silencing (37.5% → 16.4%) (Figure 6). 

## 4. Discussion

The experimental dose of doxazosin, 10–25 *μ*M, is relatively higher than the serum concentration of patients treated with doxazosin for their lower urinary tract symptoms (LUTS) [24]. Thus, doxazosin at clinical dose cannot induce significant apoptosis in the patients with prostate cancer. Therefore, the findings of new mechanisms showing the induction of apoptosis at very low concentrations of doxazosin may provide an evidence to put doxazosin as a new drug candidate for treatment of prostate cancer [25]. 

Commonly used siRNA introduction techniques are either direct introduction by transfection or introduction via plasmids that express short-hairpin RNA (shRNA) precursors of siRNA [26]. In this study, siRNA was introduced by the direct transfection way. We selected a proper oligo which could knock out Hsp27 or c-FLIP gene after 48 hours of siRNA transfection.

Rocchi et al. reported the effect of synthetic siRNA targeting Hsp27 in PC-3 and LNCaP cells. According to their reports, 1 nM of siRNA was effective to downregulate Hsp27 in mRNA and protein levels, resulting in 2.4–4-fold increase of apoptotic rates and 40%–76% inhibition of cell growth. Characteristic cleavage of caspase-3 was also observed [27].

Day et al. reported c-FLIP knockdown in MCF-7 breast cancer cells. They observed knockdown of c-FLIP gene with siRNA transfection triggering spontaneous apoptosis and inducing FADD-mediated and DR-5-mediated apoptosis. They addressed c-FLIP_L_ not c-FLIPs for having a key role in preventing spontaneous death signaling and suggested c-FLIP_L_ as a therapeutic target for breast cancer [16]. Similarly, in a report on colorectal cancer cells, Longley et al. observed that siRNA targeting c-FLIP_L_ synergistically enhanced chemotherapy-induced apoptosis [28].

In our present study, TUNEL-positive apoptotic cells increased twice with each siRNA transfection and increased over 60% after a dual silencing of 2 genes. After single gene silencing of Hsp27, apoptotic index was remarkably increased in 10 and 25 *μ*M of doxazosin treatment condition. Similarly, single silencing of c-FLIP gene enhanced doxazosin-induced apoptosis, but the degree of apoptosis was little higher in c-FLIP group compared to Hsp27 group. It can be speculated that while both factors are working as antiapoptotic factors significantly, c-FLIP plays bigger role in resisting doxazosin-induced apoptosis in PC-3 cells.

 siRNA technology can be used in combined knockdown of two genes involved in carcinogenesis or cancer progression via dual silencing. Kaulfu*β* and colleagues reported that dual silencing of insulin-like growth factor receptor and epidermal growth factor receptor resulted in an increased apoptosis rate and inhibition of cell proliferation in colorectal cancer cells. Dual silencing is technically feasible and effective in augmenting biologic effect in laboratory level. However, it has not yet been widely established. The studies using this method are limited to colorectal and breast cancers and are also limited to the region of growth factor. As far as we know, there has been no study blocking two different antiapoptotic proteins in prostate cancer cells. 

Both Hsp27 and c-FLIP have been known as strong antiapoptotic mediators. While c-FLIP manifests its role mainly in extrinsic apoptotic pathway, Hsp27 does mainly in mitochondrial pathway. For this reason, we planned to knock out these 2 factors which have different antiapoptotic mechanisms together. We observed much more amount of apoptotic bodies when these 2 factors are blocked together by siRNA technology than individual silencing of Hsp27 or c-FLIP alone. Furthermore, this effect could be seen in low concentration of 1 *μ*M of doxazosin. These show that PC-3 cells which could have resisted against apoptosis with the help of 2 survival factors became susceptible to doxazosin treatment when Hsp27 and c-FLIP are effectively knocked down together. If applied to clinical situations, this result suggests that multiple block of several antiapoptotic factors which are overexpressed and helped cancer cells to resist to treatment induced apoptosis can augment therapeutic effect even in very low concentration of the drug.

 We also observed that both factors interacted with each other. c-Flip knockout downregulated the expression of Hsp27, and similarly, silencing against Hsp27 decreased the expression of c-FLIP in RT-PCR study. These findings suggest that antiapoptotic functions of c-FLIP and Hsp27 are closely related even when the main pathways are different. We previously reported that siRNA targeting androgen receptor reversed the expression of Hsp27, GRP78, clusterin, and c-FLIP in long-term cultured androgen-independent LNCaP cell lines [28, 29]. Through these observations, we can speculate that prostate cancer cells have their own peculiar features of antiapoptotic mechanisms that include close interaction between androgen receptor and several survival factors.

## 5. Conclusions

Dual silencing is technically feasible, and dual silencing of c-Flip and Hsp27 enhances apoptosis even under 1 *μ*M, rather than low concentration, of doxazosin in PC-3 cells. This suggests a new strategy of multiple knockout of antiapoptotic and survival factors in the treatment of late-stage prostate cancer refractory to conventional therapy. We also preliminarily observed that there was interaction between c-FLIP and Hsp27 expression. Further studies revealing detailed interactions between important survival factors and androgen receptor can make another basis in reinforcing therapeutic armaments combating fatal advanced prostate cancer.
